# Barley Rhizosphere Microbiome Transplantation – A Strategy to Decrease Susceptibility of Barley Grown in Soils With Low Microbial Diversity to Powdery Mildew

**DOI:** 10.3389/fmicb.2022.830905

**Published:** 2022-05-24

**Authors:** Nina Bziuk, Lorrie Maccario, Søren J. Sørensen, Adam Schikora, Kornelia Smalla

**Affiliations:** ^1^Julius Kühn Institute (JKI) – Federal Research Centre for Cultivated Plants, Institute for Epidemiology and Pathogen Diagnostics, Braunschweig, Germany; ^2^Section of Microbiology, Copenhagen University, Copenhagen, Denmark

**Keywords:** *Hordeum vulgare*, *Blumeria graminis* f. sp. *hordei*, plant health, microbial community, rhizosphere, microbial transplant, decreased susceptibility

## Abstract

Beneficial bacteria in the rhizosphere are known to trigger faster and stronger plant immune responses to biotic and abiotic stressors. In the present study, we aimed to test the hypothesis that a rhizosphere microbiome transplant (RMT) may improve the immune response and reduce the disease rates of barley (*Hordeum vulgare*). This hypothesis was tested in a greenhouse system with the powdery mildew-causing fungus *Blumeria graminis* f. sp. *hordei* (*Bgh*). Detached rhizosphere microbiome from barley grown in a field soil was transplanted to barley seedlings grown in potting soil with reduced microbial diversity. Saline-treated plants served as control. At the three-leaf stage, barley was infected with *Bgh*. Decreased susceptibility to *Bgh* was observed for barley treated with the RMT as displayed by lower *Bgh* pustule counts in a detached leaf assay. A trend toward enhanced relative transcript abundances of the defense-related genes *PR1b* and *PR17b* was observed in leaves, 24 h after the *Bgh* challenge, when compared to the control. Moreover, 10 days after the *Bgh* challenge, the barley rhizosphere microbiome was harvested and analyzed by sequencing of 16S rRNA gene amplicons. The microbial community composition was significantly influenced by the RMT and displayed higher microbial diversity compared to the control. Furthermore, microbial beta-diversity and predicted functional profiles revealed a treatment-dependent clustering. Bacterial isolates from the RMT showed *in vitro* plant beneficial traits related to induced resistance. Our results showed that transplantation of a rhizosphere microbiome could be a sustainable strategy to improve the health of plants grown in potting soil with low microbial diversity under greenhouse conditions.

## Introduction

Plants together with their associated microorganisms build the plant holobiont (Vandenkoornhuyse et al., 2015). The genomes of these associated microorganisms are often called the second genome of the plant (Berendsen et al., 2012), as they encode important plant-related functions involved in the nutrient supply or protection against biotic and abiotic stressors (Vandenkoornhuyse et al., 2015; Berg et al., 2021). In the rhizosphere, the small zone of soil influenced by the plant root (Hiltner, 1904; Hartmann et al., 2008), the microbiome is shaped by many factors such as soil type, plant species and developmental stage, agricultural management, and geographic location (Gomes et al., 2001; Berg and Smalla, 2009; Alegria Terrazas et al., 2020; Bziuk et al., 2021).

Beneficial microorganisms in the rhizosphere can have plant growth-promoting (PGP) activity or direct antagonistic activity against soil-borne pathogens, or they can induce resistance in foliar tissues to prepare the plant for a potential future pathogen attack (reviewed by Mendes et al., 2013; Pieterse et al., 2014; Berg et al., 2017). The plant’s immune system can be enhanced by microbial secondary metabolites, such as siderophores, *N*-acyl homoserine lactones (AHLs), 2,4-diacetylphloroglucinol (DAPG), or the phytohormone indole-3-acetic acid (IAA) (Pieterse et al., 2014; Mauch-Mani et al., 2017; Gouda et al., 2018; Vannier et al., 2019). Beneficial microbes are considered important alternatives to chemical plant protection strategies for the improvement of plant performance and health in agricultural systems (Syed Ab Rahman et al., 2018). In terms of the enhanced plant defense response, a plethora of studies already reported positive effects of beneficial strains on several plant species (reviewed in Mauch-Mani et al., 2017; Syed Ab Rahman et al., 2018; Berg et al., 2021). Indeed, there are already commercially available products (e.g., Mülner et al., 2020). For barley (*Hordeum vulgare*), the model crop used in the present study, several studies reported an enhanced plant defense response triggered by AHL-producing bacteria (Han et al., 2016; Shrestha et al., 2019; Wehner et al., 2019).

Nowadays, not only single microbial biocontrol strains are used to improve plant performance and health, but also microbial consortia. The use of microbial consortia or synthetic communities (SynComs) gained attention during the last decade as promising alternatives to chemical pesticides and fertilizers (Sarma et al., 2015; Syed Ab Rahman et al., 2018; Vannier et al., 2019), and it is assumed that the enhancement of plant resistance is improved compared to single microbial inoculants (Woo and Pepe, 2018; Niu et al., 2020). Microbial consortia can enhance a plant’s resistance against biotic stressors, for instance bacterial (Gu et al., 2020) or fungal pathogens (Niu et al., 2017; Carrión et al., 2019), but they also confer tolerance toward abiotic factors like cold stress (Moradtalab et al., 2020). Furthermore, the plant itself can select a beneficial microbial consortium by recruitment of specific microbes as reported in a study on Arabidopsis leaf challenge with downy mildew-causing Oomycete *Hyaloperonospora arabidopsidis* (Berendsen et al., 2018). The challenge led to a proliferation of a consortium in the rhizosphere consisting of *Microbacterium*, *Stenotrophomonas*, and *Xanthomonas*, and the following generation of plants grown in this soil showed enhanced resistance to the disease compared to previously unplanted bulk soils (Berendsen et al., 2018). Additionally, the selection for a beneficial rhizosphere microbial community was shown to depend on the level of plant resistance (Mendes et al., 2018).

One of the best described natural microbiome-based plant defenses is suppressive soils (reviewed by Schlatter et al., 2017). Suppressive soils can inhibit the growth and activity of soil-borne pathogens due to a specific structure of the microbial community. A conducive soil can become suppressive by soil inoculation of 1–10% of a suppressive soil to the conducive soil (Schlatter et al., 2017). Moreover, soil inoculation was also reported to confer an aboveground resistance in chrysanthemum plants against thrips (Pineda et al., 2020). In the case of aboveground pests and phytopathogens, it was shown that the plants’ immune response additionally varies depending on the soil used as growth substrate (Chialva et al., 2018; Bziuk et al., 2021). In a previous study, we investigated the immune response in barley grown in a field *vs.* potting soil to *Blumeria graminis* f. sp. *hordei* (*Bgh*). We observed that barley grown in autoclaved or non-autoclaved potting soils showed considerably higher *Bgh* challenge rates than those grown in field soils, which made us assume that the rhizosphere microbiome might play an important role as a driver for better plant performance (Bziuk et al., 2021). However, soils used for barley growth differed also in soil texture and physicochemical properties (Bziuk et al., 2021), therefore, the exact role of the microbiome remained unclear.

**FIGURE 1:**
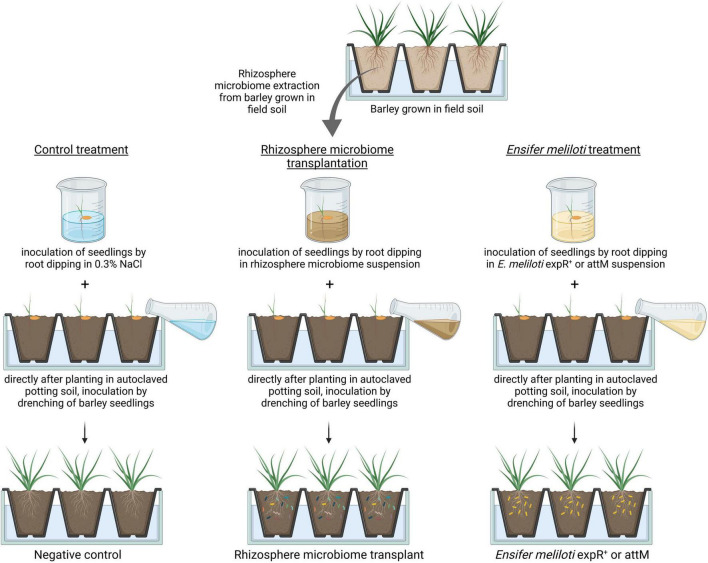
Experimental design for rhizosphere microbiome transplantation. The barley cultivar “Golden Promise” was grown in field soil under greenhouse conditions. At the three-leaf stage (BBCH13), the rhizosphere microbiome was harvested and a rhizosphere microbiome suspension was prepared. Barley seedlings were dipped into the rhizosphere microbiome suspension, planted into autoclaved potting soil, and then additionally drenched with the rhizosphere microbiome suspension. Dipping of seedlings prior to drenching was as well performed for control plants with saline (0.3% NaCl) and for plants treated with either of the two *Ensifer meliloti* strains expR^+^ and attM. The figure was created with biorender.com.

To exclude the influence of different soil physicochemical properties mentioned above (Bziuk et al., 2021), we inoculated barley seedlings grown in autoclaved standard potting soil with the rhizosphere microbiome transplant (RMT) from barley grown in field soil (Figure 1). Plants treated with saline served as a negative control. In addition, barley was inoculated with *Ensifer meliloti* Rm2011 expR^+^ (M. McIntosh; further called *E. meliloti* expR^+^), producing the AHL molecule *N*-3-oxo-tetradecanoyl-*L*-homoserine lactone oxo-C14-HSL (Zarkani et al., 2013). This strain was already shown to induce resistance against *Bgh* in barley, in an AHL-dependent manner (Hernández-Reyes et al., 2014; Shrestha et al., 2019). As a control for inoculation with *E. meliloti* expR^+^, we used the *E. meliloti* Rm2011 pBBR2attM strain (Zarkani et al., 2013; further called *E. meliloti* attM), which carries a plasmid with the lactonase gene *attM* originating from *Agrobacterium tumefaciens* that hydrolyzes the lactone ring of AHL molecules (Zarkani et al., 2013). To evaluate the plant’s immune response, we infected barley with the powdery mildew-causing fungus *Bgh*. We investigated the relative transcript abundance of the defense-related genes *PR1b* and *PR17b* in leaves by qRT-PCR and determined the *Bgh* pustule number in a detached leaf assay. Further, we examined the rhizosphere microbial community sampled 10 days after *Bgh* challenge based on 16S rRNA gene amplicon sequencing in terms of composition and predicted functions. Furthermore, bacterial isolates obtained from the RMT were characterized *in vitro* for potential plant beneficial functions. Our results strengthen the notion that microbial diversity is an important factor shaping the plant’s defense response.

## Materials and Methods

### Preparation of the Rhizosphere Microbiome Transplant

Barley (*Hordeum vulgare*) cultivar Golden Promise seeds (Simpsons Malt Limited, Berwick-upon-Tweed, United Kingdom) were germinated on wet filter paper for 3 days. Seedlings (one per pot) were planted in approximately 100 g of sieved (2 mm) field soil. Plants were further grown under greenhouse conditions at 18°C and 16/8 h (day/night) photoperiod and watered with tap water according to demand. The field soil used for this experiment was taken from a long-term field experiment in Bernburg, Germany. Agricultural management previously applied was conventional moldboard plow (20–30 cm depth), additionally to a standard N-fertilization of 200 kg/ha N and fungicide application (Deubel et al., 2011; Bziuk et al., 2021). In the three-leaf stage (BBCH13; according to Meier, 2018), rhizospheres were harvested with 0.3% NaCl as described in a later section. Rhizosphere suspensions were pooled and diluted in a ratio of 1:2 with 0.3% NaCl (saline) to establish the RMT. Four replicates of rhizospheres were additionally sampled to extract total community DNA for 16S rRNA gene amplicon sequencing as described in a later section.

### Soaking and Drenching of Barley With the Rhizosphere Microbiome Transplant

Barley seeds were pre-germinated as described above, and the seedlings were soaked for a few seconds in the RMT. Afterward, they were planted into approximately 100 g of autoclaved (3 times at 134°C for 10 min with always 1 day in between to reduce microbial diversity) potting soil (Fruhstorfer Erde, Hawita Gruppe GmbH, Vechta, Germany) 1:2 (w/w) mixed with sand (Sahara Spielsand, WECO GmbH, Leer, Germany). After planting, barley seedlings were additionally drenched with 10 ml RMT. Control plants were treated as described above with 0.3% NaCl (saline; Figure 1). Four unplanted pots drenched with 10 ml RMT or 0.3% NaCl (saline) served as bulk soil control. A dilution series of the RMT was plated on Reasoner’s 2A agar (R2A) medium supplemented with 100 μg/ml cycloheximide to reduce fungal growth, and the colony forming units (CFUs) were determined after 24 and 48 h and 6 days.

### Soaking and Drenching of Barley With *Ensifer meliloti* attM and expR^+^

Additionally to the treatments of RMT and 0.3% NaCl control, barley plants were inoculated with the resistance-inducing strain *E. meliloti* expR^+^ (Figure 1; Hernández-Reyes et al., 2014; Shrestha et al., 2019). As a control for this additional treatment, the *E. meliloti* strain attM was used, which expresses a lactonase (AttM from *A. tumefaciens*) that hydrolyzes the AHL molecule oxo-C14-HSL, the base for *E. meliloti* expR^+^-induced resistance in barley.

Overnight cultures of *E. meliloti* attM and expR^+^ in tryptone yeast (TY) medium were diluted to an OD_600_ of 0.6 and grown until an OD_600_ of 0.8 for the best AHL production. The cell cultures were centrifuged at 10,000 × *g* for 10 min and washed with 0.3% NaCl. The bacterial cells were resuspended with 0.3% NaCl to an OD_600_ of 0.1 corresponding to 10^8^ CFU/ml. Barley seedlings were treated with either of the two bacterial inocula described above. A dilution series of the inocula were plated on TY agar medium supplemented with 250 μg/ml streptomycin for *E. meliloti* expR^+^ and with 250 μg/ml streptomycin and 100 μg/ml kanamycin for *E. meliloti* attM to control the inoculum CFU.

### Challenge With *Blumeria graminis* f. sp. *hordei* (*Bgh*) and Quantification of Challenge Rates

The challenge rate of *Bgh* was determined by counting macroscopically visible *Bgh* colonies, called pustules, in three independent detached leaf assays. Barley second leaves (BBCH13) were placed with the adaxial side up, on 0.8% water agar plates and positioned inside an challenge tower. The leaves were infected with approximately 250 fresh conidia of *Bgh* per cm^2^. The procedure of *Bgh* challenge was described in detail by Bziuk et al. (2021). Briefly, *Bgh* conidia were blown from already mildewed barley plants inside the challenge tower *via* pressurized air and were allowed to settle down on the plates containing the leaves for about 10 min. Counting chambers were used to determine the conidia density. The plates containing the leaves were incubated for 5 days in a growth chamber at 20°C, 16/8 h day/night photoperiod, and 60% humidity. On day 5 of incubation, *Bgh* pustules were counted on 5 cm leaf length under a binocular. The number of pustules was divided by the leaf area and normalized to the *Bgh* inoculation density. Eight replicates per treatment were used in the first repetition and 10 replicates each for the second and third repetition. Significance was tested with SAS 9.4 (SAS Institute, Cary, NC, United States), and *p* ≤ 0.05 was assumed as different. The obligate biotrophic leaf fungus *B. graminis* (DC) Speer f. sp. *hordei* EM Marchal race A6 was maintained on barley cultivar Igri.

### RNA Extraction From Leaves and Quantitative Reverse Transcription-PCR

Quantification of the plant immune response of barley to *Bgh* was performed measuring the relative transcript abundance of the defense-related genes *PR1b* and *PR17b*, commonly used as indicator genes for defense responses in barley (Tufan et al., 2017; Shrestha et al., 2019). First, whole plants were infected with *Bgh* as described above. Samples of the second and third leaves of the whole infected and uninfected barley were taken before and 24 h after *Bgh* exposure. Three replicates per treatment were taken for each of the four individual experiments. RNA was extracted from leaf samples as described by Bziuk et al. (2021). A quantitative reverse transcription (qRT)-PCR was performed for *HvUBQ60* (5′-CAGTAGTGGCGGTCGAAGTG-3′ and 5′ACC CTCGCCGACTACAACAT-3′; Gen-Bank M60175.1) and the *Pathogenesis-Related* (*PR*) genes *PR1b* (5′-GGACTACGACTACG GCTCCA-3′ and 5′-GGCTCGTAGTTGCAGGTGAT-3′) and *PR17b* (5′-CGAGGTTCCTCGACTACTGC-3′ and 5′-ATCAC ATTCAGCCTCCGAAC-3′; Shrestha et al., 2019). The relative transcript abundance of *PR1b* and *PR17b* was normalized to *HvUBQ60* expression and the 0 h time point resulting in the 2^–ΔΔ^ ^CT^ value (Livak and Schmittgen, 2001). Significance was tested with Student’s *t*-test, and *p* ≤ 0.05 was assumed as different.

### Rhizosphere and Bulk Soil Sampling and Processing

Four replicate plants of barley grown in field soil were used for RMT preparation (described above), as well as four replicate plants grown in potting soil (10 days after exposure to *Bgh*) for each treatment (RMT or control, *Bgh*-infected, or uninfected), were used for rhizosphere sampling. First, loosely attached soil was removed by shaking the plant, additionally, a tweezer was used to remove bigger potting soil particles. Afterward, the complete root system with more tightly bound soil was transferred to a 50 ml tube and nine volumes of 0.85% NaCl were added. After 1-min vortexing to detach the rhizosphere the roots were removed. Rhizosphere suspensions were centrifuged for 20 min at 8,500 *g* and the supernatant was discarded. Bulk soil samples were taken from the respective unplanted pots by mixing the soil carefully. Rhizosphere and bulk soil pellets were stored at –20°C until total community DNA extraction.

### Total Community DNA Extraction and 16S rRNA Gene Amplicon Library Preparation

For total community DNA extraction, 0.5 g (wet weight) of barley rhizosphere or bulk soil samples were treated with the FastDNA Spin Kit for Soil (MP Biomedicals, Eschwege, Germany) according to the manufacturer’s instructions.

Amplicon sequencing libraries of rhizosphere and bulk soil samples were prepared in a two-step PCR targeting the V3-V4 region of the 16S rRNA gene. The first PCR was performed using the Uni341F (5′-CCTAYGGGRBGCASCAG-3′) and Uni806R (5′-GGACTACHVGGGTWTCTAAT-3′) primers (Yu et al., 2005; Caporaso et al., 2011; Sundberg et al., 2013) as described by Bziuk et al. (2021). First PCR products were purified using the HighPrep PCR clean-up (MagBio Genomics, Gaithersburg, MD, United States) in a 0.65:1 (beads:PCR reaction) volumetric ratio. The second PCR was used to add Illumina sequencing adapters and sample-specific dual indices (IDT Integrated DNA Technologies, Coralville, IA, United States) using PCRBIO HiFi (PCR Biosystems Ltd., London, United Kingdom) for 15 amplification cycles. Second PCR products were purified as described above, and further processing of the purified PCR products was performed as described in the study by Bziuk et al. (2021). The resulting library was sequenced on an Illumina MiSeq platform at the Section of Microbiology, University of Copenhagen (Denmark) using Reagent Kit v2 (2 × 250 cycles) (Illumina, San Diego, CA, United States) and including 1st and 2nd PCR negative controls, as well as a mock control.

### Generation of Amplicon Sequence Variants

Amplicon sequencing data analysis was performed as previously described in the study by Bziuk et al. (2021). Briefly, primer sequences were removed using Cutadapt v.2.3 (Martin, 2011), and amplicon sequence variants (ASV) were generated using the DADA2 version 1.10.0 (Callahan et al., 2016) plugin for QIIME2 (Bolyen et al., 2019) and using q2-feature-classifier classify-sklearn module trained with SILVA SSU rel. 132 database (Quast et al., 2013).

The ASV dataset was further cleaned using the package phyloseq (McMurdie and Holmes, 2013) of RStudio (RStudio, Boston, MA, United States) version R3.6.3. ASV affiliated to chloroplasts (28 ASV representing 0.986% of total reads), mitochondria (four ASV representing 0.289% of total reads), and unassigned Kingdom or Phylum (30 ASV representing 0.048% of total reads) were removed from the dataset. The dataset was also filtered based on the 16S rRNA V3-V4 region size keeping only ASV of a length between 350 and 440 bp (21 ASV were removed). DECONTAM (Davis et al., 2018) was used to identify potential contaminations as determined by the prevalence of ASV in the negative controls (from 1st and 2nd PCR) revealing no true contaminants. After cleaning, 97.67% of the dataset was kept. Rhizosphere samples obtained a median of 12 204 reads and 323.5 ASV per sample. Bulk soils contained a median of 7470.5 reads and 271 ASV per sample.

### Sequence Data Analysis

Alpha-diversity indices were calculated based on read count data 100 times randomly subsampled to the lowest number of sequences (6946) using RStudio R3.6.3 packages multcomp (Hothorn et al., 2008) and vegan (Oksanen et al., 2019). Three samples were excluded from the analysis due to a low amount of sequence reads. Analysis of variance (ANOVA) and permutational analysis of variance (PERMANOVA) was performed with vegan package and based on read count data, the principle component analysis (PCA) based on relative abundances. PERMANOVA was conducted based on Bray–Curtis dissimilarity indices (10,000 permutations). Treatment-dependent changes in the relative abundances of microbial genera were examined using the packages edgeR (Robinson et al., 2010) and phyloseq (McMurdie and Holmes, 2013) with a likelihood ratio test under negative binomial distribution and generalized linear models (FDR-corrected; *p* ≤ 0.05). The heatmap visualizing relative abundances of selected genera was created using the pheatmap package (Kolde, 2015). Functional predictions of the microbial community based on the 16S rRNA amplicon data were performed using Tax4Fun2 (Wemheuer et al., 2020) with the Ref99NR reference dataset.

### Cultivation and Characterization of Fast Growing Members of the Rhizosphere Microbiome Transplant

Bacterial isolates were randomly picked from plates with 20–100 colonies obtained after plating a serial dilution of the RMT on the R2A medium and incubation at 28°C for 6 days. Genomic DNA was extracted from 10 representative RMT isolates using the Genomic DNA Extraction Kit (Qiagen, Hilden, Germany) and the Silica Bead DNA Gel Extraction Kit (Thermo Fisher Scientific, Waltham, MA, United States). A BOX-PCR fingerprint was conducted for each isolate (Martin et al., 1992) and taxonomic affiliation was determined based on sequencing (Macrogen Europe B.V., Amsterdam, Netherlands) of a partial 16S rRNA gene fragment (Heuer et al., 2009). The sequences were trimmed and assembled using CLC MainWorkbench 20.0.3 (Qiagen, Aarhus, Denmark) and taxonomic affiliation was determined based on the NCBI database (NCBI, Bethesda, MD, United States).

Bacterial isolates of the RMT were tested *in vitro* for potential plant beneficial activities: protease, β-1,3-glucanase, and cellulase (Weinert et al., 2010), as well as chitinase activity (Berg et al., 2001). Further assays tested for ACC deaminase and IAA production (Koo et al., 2010), production of siderophores (Schwyn and Neilands, 1987), and AHLs using the sensor strains *Chromobacterium violaceum* VIR07 and cv026 (Durán et al., 2016).

## Results

### Decreased *Bgh* Challenge Rate of Barley After Rhizosphere Microbiome Transplantation

**FIGURE 2:**
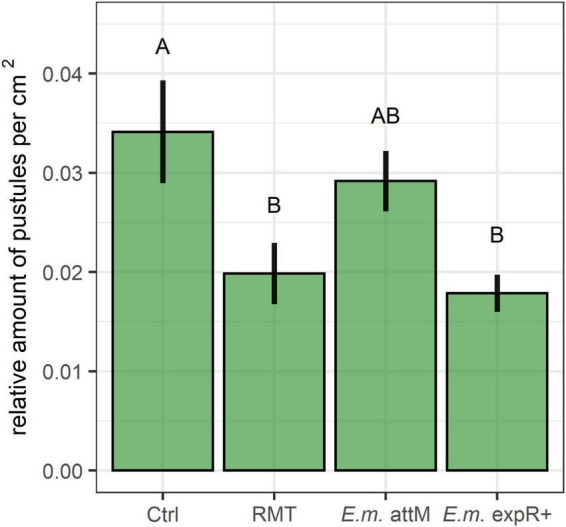
*Bgh* challenge rate decreased after rhizosphere microbiome transplantation. Effects of inoculation on the relative amount of *Bgh* pustules per cm^2^ were determined in a detached leaf assay. Barley was either treated with the rhizosphere microbiome transplant (RMT) from field soil or saline (0.3% NaCl) as control (Ctrl). Inoculation of the *Ensifer meliloti* (*E.m*.) strains expR^+^ (resistance inducing) and attM (control) was used as internal positive control. Bars represent mean ± SE of four independent experiments (*n* = 10, except for the first repetition *n* = 8). Different letters indicate significant differences (Tukey’s test; *p* ≤ 0.05).

The challenge rate determined in the detached leaf assay by *Bgh* pustule counts revealed significantly decreased counts for RMT-treated barley compared to the control (Figure 2). As an internal control, we used *E. meliloti* expR^+^ and attM strains, as described in the section “Materials and Methods.” Plants treated with *E. meliloti* expR^+^ showed a decreased challenge rate compared to *E. meliloti* attM. Although the decreased challenge rate of *E. meliloti* expR^+^ was not significant compared to *E. meliloti* attM when all treatments were statistically analyzed together (Tukey’s test; Figure 2), it was highly significant in a single comparison with *E. meliloti* expR^+^ *vs.* attM (Student’s *t*-test, *p* = 0.003). *Bgh* challenge rates revealed similar levels for RMT-treated barley and the internal positive control *E. meliloti* expR^+^.

**FIGURE 3:**
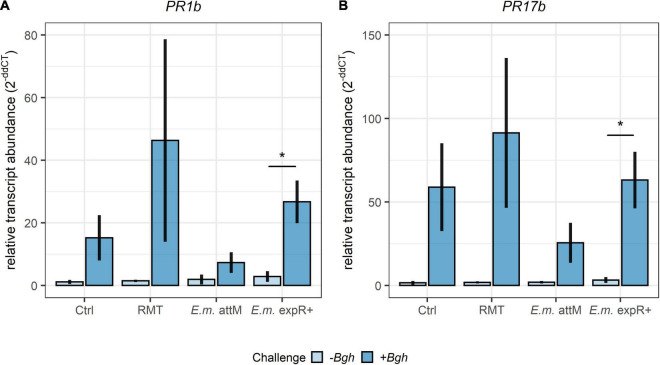
Higher relative transcript abundances of defense-related genes after *Bgh* challenge of barley treated with the rhizosphere microbiome transplant. Relative transcript abundances measured by qRT-PCR of the defense-related genes **(A)** *PR1b* and **(B)** *PR17b* in barley leaves 24 h after *Bgh* challenge. Barley was treated with the rhizosphere microbiome transplant (RMT), saline (0.3% NaCl) as control (Ctrl) or either of the two *E. meliloti* (*E.m*.) strains expR^+^ and attM. Bars represent the mean ± SE of three individual experiments (with each *n* = 3 per treatment). Transcript abundance was normalized to *UBQ* transcript abundance and the 0 h time point. Significant differences were tested by Student’s *t*-test (*p* ≤ 0.05).

Relative transcript abundances of the two defense-related genes *PR1b* and *PR17b* showed similar levels for the uninfected plants in all treatments (Figures 3A,B). After the *Bgh* challenge, relative transcript abundances of *PR1b* and *PR17b* were enhanced in all treatments investigated, but only *E. meliloti* expR^+^ treated samples displayed significantly higher relative transcript abundance for both genes. Barley plants that had received the RMT showed higher relative transcript abundances compared to the control, although not significant due to the high variability between the individual experiments.

### Microbial Diversity and Community Composition Were Highly Influenced by the Rhizosphere Microbiome Transplantation

The microbial diversity and community composition of barley rhizosphere were analyzed across treatments [i.e., rhizosphere (RS), RMT-treated and control (Ctrl), either *Bgh*-infected (+*Bgh*) or uninfected (–*Bgh*), bulk soil (BS) and field rhizosphere], and a total of 330,701 reads were obtained that assigned to 3779 ASV. However, three samples from different treatments had to be excluded from the analysis due to low read numbers (RS Ctrl, BS Ctrl, BS RMT). The rarefaction curve of the remaining samples (Supplementary Figure 1) showed a sufficient library size to cover the microbial diversity.

**FIGURE 4:**
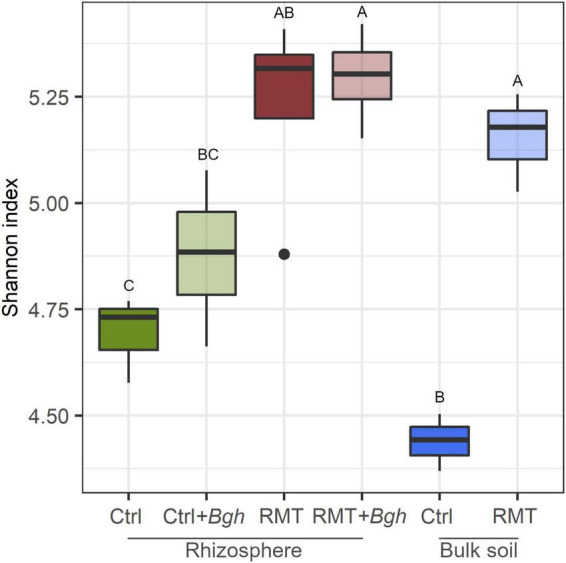
Microbial alpha-diversity is higher after rhizosphere microbiome transplantation. Shannon index of rhizosphere microbiome transplant (RMT) or control (Ctrl) of barley rhizosphere and bulk soil samples. Barley was additionally infected with *Bgh*. Different letters indicate significant differences (Tukey’s test *p* ≤ 0.05) that were determined for rhizosphere and bulk soil samples independently. Means ± SD value are written in Supplementary Table 1.

The RMT treatment increased microbial diversity in barley rhizosphere and in bulk soil, compared to the control (Figure 4 and Supplementary Table 1). For instance, Species richness was 1.5 × higher in RMT-treated bulk soil than in the control soil (Supplementary Table 1). Aboveground *Bgh* exposure did not affect the Species richness or Shannon diversity in the rhizosphere of barley treated with RMT or the control. ANOVA of alpha-diversity indices revealed a significant influence of the RMT treatment on both Species richness and Shannon diversity (*p* ≤ 0.001). Pielou’s evenness was also affected by RMT (*p* ≤ 0.001) with higher evenness for RMT-treated samples (Supplementary Table 1).

**FIGURE 5:**
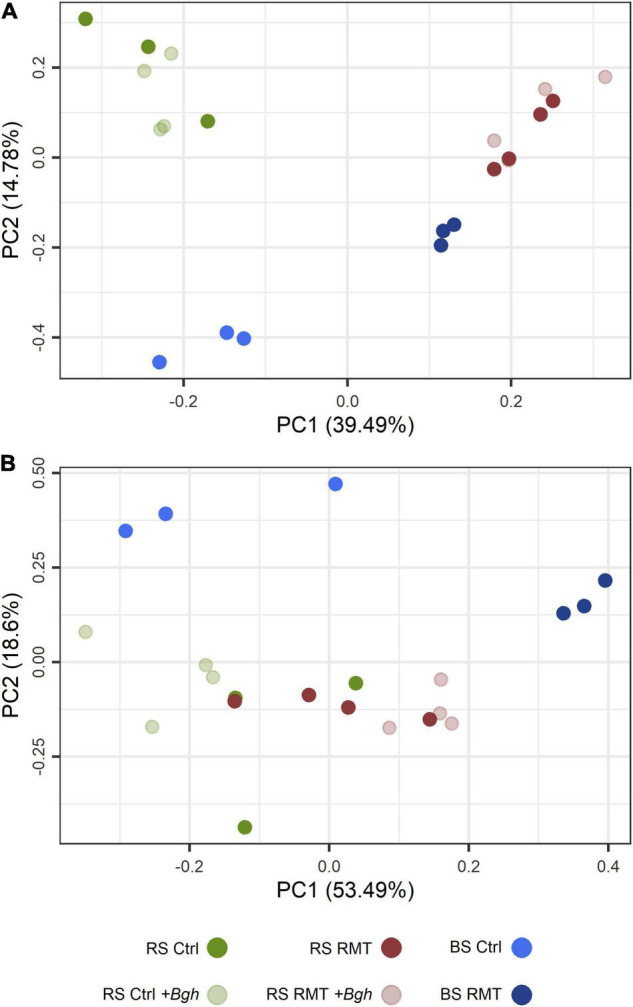
Community composition and predicted functional profile of barley rhizosphere and bulk soil microbial communities. **(A)** Microbial community composition of barley rhizosphere (RS) and bulk soil (BS) was visualized by constrained principal component analysis (PCA) based on the relative abundances of rhizosphere microbiome transplant (RMT) and control (Ctrl) amplicon sequence variants (ASV), either infected with *Bgh* or not. **(B)** Predicted KEGG Orthologs (KO) for each microbial community of RS or BS, treated with either RMT or as Ctrl with and without *Bgh* infected, visualized by PCA based on the relative abundance of each predicted KO.

Visualization of the microbial beta-diversity by PCA (Figure 5A) revealed a clear separation along the first axis into RMT and control, for both rhizosphere and bulk soil samples. Along the second axis, the microhabitats rhizosphere and bulk soil were separated. There was no clear distinction between barley infected with *Bgh* or not for both RMT and control rhizospheres, as the respective samples clustered together.

The effect of the RMT treatment on the microbial community composition was verified by PERMANOVA (*R*^2^ = 0.46; *p* ≤ 0.001; Table 1), as well as the effect of the microhabitat (*R*^2^ = 0.13; *p* ≤ 0.001). *Bgh* challenge did not affect the microbial community composition in the rhizosphere of RMT-treated barley (*R*^2^ = 0.17; *p* = 0.22), but interestingly, *Bgh* infected significantly influenced the control (*R*^2^ = 0.27; *p* ≤ 0.05).

**TABLE 1:** PERMANOVA of barley rhizosphere (RS) and bulk soil (BS) samples, either treated with rhizosphere microbiome transplant (RMT) or as control (Ctrl).

PERMANOVA	*R* ^2^	*p* ≤
Microhabitat RS *vs.* BS	0.13	0.001
Rhizosphere Ctrl *vs.* RMT	0.57	0.001
RS Ctrl –*Bgh vs*. +*Bgh*	0.27	0.05
RS RMT –*Bgh vs.* +*Bgh*	0.17	0.22

*Barley was additionally infected with Bgh (+Bgh) or not (–Bgh).*

### Taxonomic Microbial Community Composition Shaped by Rhizosphere Microbiome Transplant and, to a Lesser Extent, by *Bgh* Challenge

Dominant phyla in the rhizosphere and bulk soil samples of both RMT and control samples were Proteobacteria, Bacteroidetes, Firmicutes, and Actinobacteria (Table 2). Notable differences in relative abundances between RMT and control samples were detected in the rhizosphere of uninfected and *Bgh*-infected barley for Firmicutes that decreased after transplantation (RMT 9.0/6.5% *vs.* Ctrl 17.2/15.5%) and Actinobacteria that increased after transplantation (RMT 6.4/6.5% *vs.* Ctrl 1.3/2.8%). Furthermore, increased relative abundance after RMT treatment was also observed for the lower abundant Verrucomicrobia (RMT 2.4/1.3% *vs.* Ctrl 1.1/1.4%), as well as for Patescibacteria (RMT 2.2/3.7% *vs.* Ctrl 0.1/0.1%). Verrucomicrobia was the only phylum that also displayed significant differences for RMT-treated and *Bgh*-infected barley rhizosphere (–*Bgh* 2.4% *vs.* +*Bgh* 1.3%).

**TABLE 2:** Relative abundances of microbial phyla present in barley rhizosphere and bulk soil samples, either treated with rhizosphere microbiome transplant (RMT) or as control (Ctrl).

	Rhizosphere				Bulk soil	
		
	Ctrl		RMT		Ctrl	RMT
		
	–*Bgh*	+*Bgh*	–*Bgh*	+*Bgh*		
Proteobacteria	57.91^a^	54.92^a^	53.16^a^	58.61^a^	59.81^a^	63.06^a^
Bacteroidetes	22.35^a^	25.15^a^	26.21^a^	22.85^a^	27.77^a^	24.26^b^
Firmicutes	17.15^a^	15.51^ab^	8.98^bc^	6.47^c^	8.15^a^	5.44^a^
Actinobacteria	1.33^b^	2.84^b^	6.35^a^	6.45^a^	2.77^a^	2.78^a^
Verrucomicrobia	1.11^b^	1.36^b^	2.43^a^	1.29^b^	1.41^a^	2.06^a^
Patescibacteria	0.11^b^	0.10^b^	2.22^ab^	3.73^a^	0.00^a^	1.54^a^
Rare phyla (≤ 1%)	0.03	0.12	0.36	0.29	0.07	0.39

*Barley was additionally infected with Bgh (+Bgh) or not (–Bgh). Different letters indicate significant differences (Tukey’s test p ≤ 0.05), rhizosphere and bulk soil samples were statistically analyzed separately.*

**FIGURE 6:**
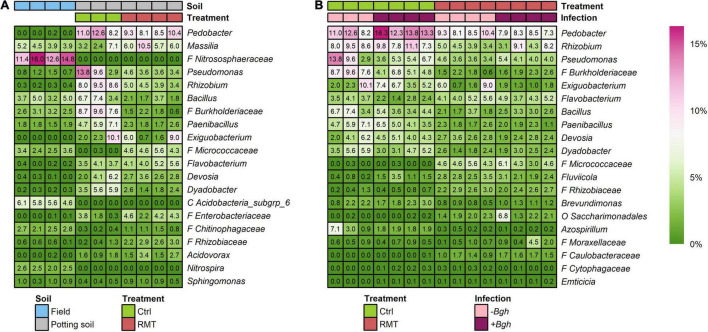
Relative abundances of taxa. **(A)** Abundant taxa present in the rhizosphere of barley grown in field soil representing the rhizosphere microbiome transplant (RMT), rhizosphere of RMT-treated and control (Ctrl) plants grown in potting soil with reduced microbial diversity. **(B)** Abundant taxa differing between Ctrl and RMT rhizospheres as determined with a likelihood ratio test under negative binomial distribution and generalized linear models (FDR-corrected; *p* ≤ 0.05). Additionally, relative abundances of *Fluviicola*, *Emticicia*, and *Cytophagaceae* were differing due to *Bgh* challenge in Ctrl samples. F, family; O, order; C, class.

Some highly abundant taxa present in the rhizosphere of barley grown in field soil representing the RMT were not able to establish in the rhizosphere of barley grown in potting soil: *Nitrososphaeraceae*, Acidobacteria subgroup 6 and *Nitrospira* (Figure 6A). Other taxa were well transferred by RMT, like *Chitinophagaceae* or *Sphingomonas*, as these taxa had similar relative abundances in both barley rhizosphere from field and RMT-treated, whereas they were less abundant in the rhizosphere of control plants (Figure 6A). Some taxa that were highly abundant in the rhizosphere from barley grown in field soil that served as RMT had similar relative abundances in the RMT-treated rhizosphere, such as *Bacillus* or *Paenibacillus*, but were even more abundant in the control.

Several taxa were highly abundant in both rhizosphere and bulk soil samples of all potting soil samples (RMT/Ctrl), such as *Pedobacter*, *Rhizobium*, *Massilia*, or *Pseudomonas*. The RMT treatment influenced the relative abundances of several highly abundant taxa (Figure 6B and Supplementary Table 3): unclassified members of *Micrococcaceae* and *Rhizobiaceae* were significantly higher in relative abundance for both uninfected and *Bgh*-infected samples in the rhizosphere of RMT-treated barley, whereas in the control *Burkholderiaceae*, *Paenibacillus*, *Dyadobacter*, and *Azospirillium* had higher relative abundances. *Fluviicola* showed higher relative abundance in the rhizosphere of uninfected RMT-treated barley compared to the control, whereas for uninfected control rhizosphere samples *Rhizobium*, *Pseudomonas*, *Bacillus*, and *Brevundimonas* displayed higher relative abundances. The influence of RMT in *Bgh*-infected rhizosphere samples revealed a higher relative abundance of *Flavobacterium*, *Saccharimonadales*, and *Moraxellaceae* compared to the control. For the *Bgh*-infected control plants, *Pedobacter*, *Exiguobacterium*, and *Devosia* had higher relative abundances compared to the respective RMT samples.

Interestingly, the unclassified members of *Micrococcaceae* that were highly abundant in RMT-treated rhizospheres (4.8%), were almost not detected in the control (0.1%). The relative abundance of *Micrococcaceae* in barley rhizosphere from field soil that served as RMT was about 3.0%. The respective sequences of the unclassified *Micrococcaceae* ASV were blasted to the NCBI database for further taxonomic affiliation. All eight ASV belonged to either *Arthrobacter* or *Pseudarthrobacter* (Supplementary Table 2).

Differences in relative abundance due to the *Bgh* challenge were not detected for RMT-treated barley. Only in the rhizosphere of the control, the three taxa were significantly increased in relative abundance after exposure to *Bgh*: *Fluviicola*, *Emticicia*, and *Cytophagaceae* (Figure 6B).

### Predicted Functional Profile of the Microbial Community Is Mainly Influenced by *Bgh* Challenge

In order to investigate the putative functions of members of the microbial communities, a functional prediction profile was established using Tax4Fun2 (Wemheuer et al., 2020). In total, 22,831 Kyoto Encyclopedia of Genes and Genomes (KEGG) Orthologs (KOs) were predicted among the different communities in rhizosphere and bulk soil samples.

Predicted functional profiles of the RMT-treated or control bulk soil communities clustered separately as revealed by PCA based on the relative abundances of predicted KOs (Figure 5B). In contrast, the predicted functional profiles for the rhizosphere of RMT and control plants were found to be similar. *Bgh* challenge seemed to influence the functional prediction profile of RMT and control rhizospheres, as observed in the PCA (Figure 5B). PERMANOVA was used to verify the differences visualized in the PCA. The predicted functional profiles were significantly different between the microhabitat rhizosphere or bulk soil (*R*^2^ = 0.20, *p* ≤ 0.001), as well as between the rhizospheres of RMT-treated and control plants (*R*^2^ = 0.40, *p* ≤ 0.001). Although the rhizospheres of uninfected RMT and control plants grouped closely together in the PCA, they were significantly different (*R^2^* = 0.43, *p* ≤ 0.05). The highest influence on the predicted functional profile was exerted by challenge with *Bgh* (*R*^2^ = 0.71, *p* ≤ 0.05).

### Bacterial Isolates From Rhizosphere Microbiome Transplant Revealed Potential Plant Beneficial Activities

**FIGURE 7:**
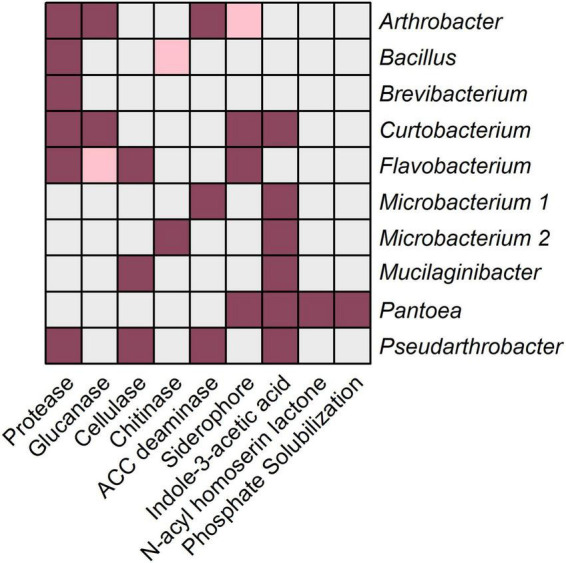
*In vitro* functional characteristics of bacterial isolates obtained from the rhizosphere microbiome transplant. Ten representative bacterial isolates obtained from the rhizosphere microbiome transplant were tested *in vitro* for their potential plant beneficial characteristics in different bioassays described in the section “Materials and Methods.” Gray color represents a negative result, pink color represents a slight positive result and violet color represents a positive result.

The prediction of functions based on 16S rRNA gene amplicon data might give an insight into the potential functional capabilities of a microbial community, but it remains unclear whether these functions are actually exhibited. Therefore, 10 randomly chosen bacterial isolates obtained from the RMT were characterized to verify their potential plant beneficial characteristics. BOX-PCR fingerprints of bacterial RMT isolates showed diverse patterns suggesting high diversity within the isolates. Among the randomly selected isolates, nine out of 10 belonged to different genera (Supplementary Table 4), based on partial 16S rRNA gene sequence annotation using the NCBI database: *Microbacterium*, *Brevibacterium*, *Pantoea*, *Arthrobacter*, *Mucilaginibacter*, *Flavobacterium*, *Pseudarthrobacter*, *Bacillus*, and *Curtobacterium*. *Bacillus*, *Flavobacterium*, and members of *Micrococcaceae* (*Arthrobacter* and *Pseudarthrobacter*) were also of higher abundance in the 16S rRNA amplicon data. Several potential plant beneficial characteristics were detected by means of *in vitro* assays (Figure 7). Protease activity and IAA production were characteristics presented by most of the isolates (6/10). Lytic enzyme activities demonstrated by several isolates were β-1,3-glucanase (*Arthrobacter*, *Curtobacterium*, and *Flavobacterium*), cellulase (*Mucilaginibacter*, *Flavobacterium*, and *Pseudarthrobacter*), and ACC deaminase (*Microbacterium*, *Arthrobacter*, and *Pseudarthrobacter*). Siderophore production was presented by *Pantoea*, *Flavobacterium*, *Arthrobacter*, and *Curtobacterium*. *Pantoea* was the only isolate showing AHL production and the ability to solubilize phosphate. *Microbacterium* was the only isolate able to degrade chitin. *Brevibacterium* and *Bacillus* showed only protease activity. In summary, representative bacterial isolates of the RMT were taxonomically diverse and showed *in vitro* potential plant beneficial properties.

## Discussion

Microbiome modulation is thought to be an efficient method to improve plant performance and health (Berg et al., 2021). In the present study, we demonstrated that transplantation of a rhizosphere microbiome from barley grown in field soil to barley grown in potting soil with reduced microbial diversity significantly decreased the susceptibility to the aboveground leaf pathogen *Bgh*. A higher belowground microbial diversity does not only suppress soil-borne pathogens or invaders as reported before (Kusstatscher et al., 2019; Schierstaedt et al., 2020) but can also decrease the susceptibility toward foliar pathogens. Our results showed that the transplantation of a rhizosphere microbiome with high microbial diversity may be an important factor shaping the plant defense response.

Numerous studies already reported on the importance of microbial diversity for belowground plant protection against pathogens, for instance in suppressive soils (reviewed by Berg et al., 2017; Schlatter et al., 2017). Although single microorganisms or synthetic communities were described to confer resistance to aboveground pathogens *via* induced systemic resistance or priming (e.g., reviewed by Mauch-Mani et al., 2017; Vannier et al., 2019), only a few studies suggested the positive impact of the whole soil or rhizosphere microbiome on resistance to aboveground phytopathogens or pests (Chialva et al., 2018; Pineda et al., 2020; Bziuk et al., 2021), however, in most cases, different soils were used. Here, we showed that microbiome modulation decreased barleys’ susceptibility to powdery mildew, excluding the influence of different physicochemical properties from diverse soils.

Although the relative transcript abundances of the defense-related genes *PR1b* and *PR17b* were increased after *Bgh* challenge in RMT-treated barley compared to the control, due to high variation between the three independent experimental repetitions, we cannot draw conclusions on their significance. However, the relative transcript abundance of those genes was significantly increased in plants inoculated with *E. meliloti* expR^+^, after infected with *Bgh.* Previous studies indicated that *E. meliloti* expR^+^-induced resistance is based on the production of the AHL oxo-C14-HSL and could be mimicked using the synthetic molecule, in a phenomenon called AHL-priming (Hernández-Reyes et al., 2014). In the latter study, the authors observed a significant increase in relative transcript abundance of *PR1b* in plants exposed to oxo-C14-HSL and infected with *Bgh* after 24 h. Here, we assessed a greenhouse-based system. In contrast to already published *Bgh* pustule counts, transcript abundance data for AHL-primed barley after the *Bgh* challenge were available only from *in vitro* systems. Compared to the *in vitro* system of Hernández-Reyes et al. (2014), our results suggest that relative transcript abundance data generated from *in vivo*, greenhouse-grown barley inoculated with *E. meliloti* might not be consistent enough, probably due to the high variability of the gene expression level in several independent experiments. Nevertheless, the trend toward higher relative transcript abundances of defense-related genes in RMT-treated barley, together with the significantly decreased *Bgh* challenge rate, provided evidence of a decreased susceptibility given by the RMT.

**FIGURE 8:**
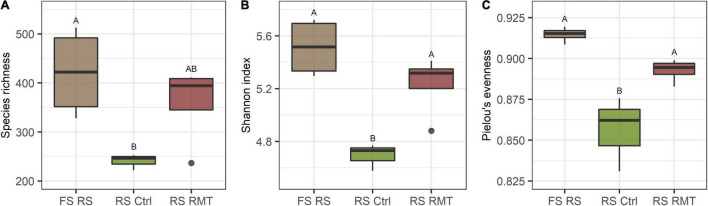
Comparison of microbial diversity between barley rhizosphere from field soil, rhizosphere microbiome transplant, and control treatments. **(A)** Species richness, **(B)** Shannon index, and **(C)** Pielou’s evenness of barley rhizosphere (RS) from field soil (FS) that served as rhizosphere microbiome transplant (RMT), additionally to rhizosphere from barley grown in potting soil treated with RMT or the control (Ctrl). Different letters indicate significant differences (Tukey’s test; *p* ≤ 0.05).

The observation that the microbial alpha-diversity increased in RMT-treated rhizosphere and bulk soil was not surprising, as a highly diverse microbiome from field soil was transferred to potting soil with reduced microbial diversity (Figure 8). The reduced number of *Bgh* pustules, combined with the trend toward increased gene expression in RMT-treated barley suggests that the plants were prepared for a potential pathogen attack. As a consequence of the modulation of the rhizosphere microbiome by RMT, no influence on the community composition was visible after the *Bgh* challenge and no recruitment of specific microbes is needed, as these might already be present. The significant influence of the *Bgh* challenge on the microbial community composition of control samples, together with the lower alpha-diversity indices in the control, suggests that the low diverse microbiome in potting soil was disturbed and on contrary, the higher diversity in the RMT-treated rhizosphere buffered the impact of the aboveground challenge. Similar observations were reported before, such as the success of an invading species in soil was linked to the community diversity and evenness (De Roy et al., 2013; Mallon et al., 2015; Schierstaedt et al., 2020). Berg et al. (2017) suggested as well that diseases are often correlated with microbial dysbiosis or reduced soil diversity. Berg et al. (2021) suggested the “Anna Karenina Principle” for the plant microbiome that was previously considered by Zaneveld et al. (2017) for the animal microbiome. The principle describes that dysbiotic plants vary more in their microbiome composition compared to healthy plants (Berg et al., 2021). Healthy plants grown in soil with comparable high microbial diversity might have better options to select plant beneficial microbes or functional traits compared to unhealthy, dysbiotic plants.

Furthermore, we detected taxa with treatment-dependent relative abundances, also in response to the *Bgh* challenge (Figure 6B). In *Bgh*-infected control samples, the genus *Fluviicola* was about four times higher compared to uninfected barley (relative abundance 1.9 *vs.* 0.5%). *Fluviicola* (*Flavobacteriales*) was first described by O’Sullivan et al. (2005). By now, the type strain is sequenced (Woyke et al., 2011), but only four species of the genus *Fluviicola* are described. There is not much knowledge of the environmental and functional role of *Fluviicola* in a microbial community, but there are reports on different contaminated soils and thus it might be involved in the degradation of the respective contaminants (Song et al., 2016; Lu and Lu, 2018; Jia et al., 2019; Revelo Romo et al., 2019). This is the first time that ASV affiliated to this genus were detected in the rhizosphere of barley infected aboveground with *Bgh* and, although not measured in the present study, it is assumed that released phenolic compounds, typically involved in plant defense, (Mandal et al., 2010) might have triggered the proliferation of *Fluviicola* sp. Other taxa with significantly higher relative abundances after the *Bgh* challenge in the rhizosphere of the control samples belonged to the family *Cytophagaceae*, whose members are able to degrade cellulose (Stanier, 1940). Degradation of cellulose seems plausible as the challenge results in increased amounts of dead plant material that can serve as a nutrient source. One member of *Cytophagaceae*, the genus *Emiticicia*, revealed higher relative abundances after *Bgh* challenge, however, not much is known about this genus (Saha and Chakrabarti, 2006). The results do not suggest taxa responsible for plant protection or microbial dysbiosis, but rather point toward increased microbial degradation.

The predicted functional profiles revealed that the different bulk soil microbial communities were also distinct in their predicted functional capabilities, likely due to the RMT treatment. In the uninfected rhizosphere (RMT/Ctrl; –*Bgh*), microbial communities were significantly different in their structure, but probably displayed similar major functions (Figure 5B). After the *Bgh* challenge (RMT/Ctrl; +*Bgh*), the predicted functional profile of rhizosphere samples showed higher separation between RMT and control (Figure 5B; PERMANOVA), suggesting a change in predicted functional capabilities after the pathogen challenge. Taken together, RMT mainly shifted the microbial community structure but the *Bgh* challenge mainly shifted the predicted functional profile.

The 16S rRNA gene amplicon sequencing method used in the present study has limitations, therefore, bacterial isolates obtained from the RMT were investigated for potential plant beneficial functions. Interestingly, some isolates belonged to highly abundant members of the RMT-treated rhizosphere, such as *Arthrobacter* and *Pseudarthrobacter* (*Micrococcaceae*). Unclassified *Micrococcaceae* displayed increased relative abundances in RMT-treated rhizosphere compared to the control based on the 16S rRNA amplicon data. The respective ASV sequences revealed high sequence similarity to the genera *Arthrobacter* or *Pseudarthrobacter* (Supplementary Table 2). *Micrococcaceae* were also highly abundant in the respective rhizosphere from field soil used for RMT (relative abundance field soil: 3.0%; Ctrl: 0.1%; RMT: 4.8%). All obtained bacterial isolates showed at least one potential plant beneficial property, nevertheless, isolates affiliated with *Pantoea*, *Curtobacterium*, as well as *Arthrobacter*, *Pseudarthrobacter*, and *Flavobacterium* displayed the highest number of potential plant beneficial properties. Strains of the genus *Arthrobacter* are already known for their plant-beneficial capabilities, for instance, the production of ACC deaminase (Barnawal et al., 2017) and siderophores (Sharma et al., 2016), as also observed in the present study. Furthermore, *Arthrobacter* species were found to enhance the salt tolerance of wheat plants (Barnawal et al., 2017; Safdarian et al., 2019) and showed antagonistic activity against several fungal plant pathogens (Pylak et al., 2020), suggesting a high potential for plant protection.

The present study showed that microbiome modulation by RMTs might be a sustainable strategy to improve the health of plants grown in low diversity soil, such as potting soils that are used for greenhouse production. Plants grown in potting soil showed higher disease incidence than previously reported for barley infected with *Bgh*. Treatment of barley grown in potting soil with RMT improved plant health by decreasing the challenge rate of *Bgh*. The rhizosphere microbiome of RMT-treated barley showed a higher microbial diversity and a higher relative abundance of potentially plant beneficial taxa such as *Arthrobacter* and *Pseudarthrobacter*. The present study provides unequivocal evidence for the important role of the rhizosphere microbiome and its natural diversity for plant health.

## Data Availability Statement

The data presented in the study are deposited in the Sequence Read Archive of NCBI, BioProject accession number: PRJNA777052 (as a reviewer, you can use this link: https://dataview.ncbi.nlm.nih.gov/object/PRJNA777052?reviewer=dm8chdu2vvu4n0aqfva7994oul).

## Author Contributions

NB and KS designed the study. NB performed the experiments and conducted the statistical analyses. SS coordinated the sequencing facility at KU. LM carried out the amplicon sequencing. LM and NB performed the bioinformatic analyses of amplicon sequencing data. NB wrote the manuscript with the contributions of KS and AS. All authors read and approved the final manuscript.

## Conflict of Interest

The authors declare that the research was conducted in the absence of any commercial or financial relationships that could be construed as a potential conflict of interest.

## Publisher’s Note

All claims expressed in this article are solely those of the authors and do not necessarily represent those of their affiliated organizations, or those of the publisher, the editors and the reviewers. Any product that may be evaluated in this article, or claim that may be made by its manufacturer, is not guaranteed or endorsed by the publisher.
