# Supplementary material for: Barley Rhizosphere Microbiome Transplantation – A Strategy to Decrease Susceptibility of Barley Grown in Soils With Low Microbial Diversity to Powdery Mildew
Source: Front Microbiol. 2022 May 24;13:830905. doi: 10.3389/fmicb.2022.830905 (PMC9173696; doi:10.3389/fmicb.2022.830905)
Supplement: Supplementary file 1 [file Data_Sheet_1.DOCX]

Supplementary Material

# Supplementary Figures and Tables

## Supplementary Figures

**Supplementary Figure 1.** Rarefaction curves of 16S rRNA gene amplicon data of barley rhizosphere (RS) and bulk soil (BS) samples after cleaning. FS: field soil; PS: potting soil; Ctrl: control; RM: rhizosphere microbiome transplant; Bgh: challenge with Bgh


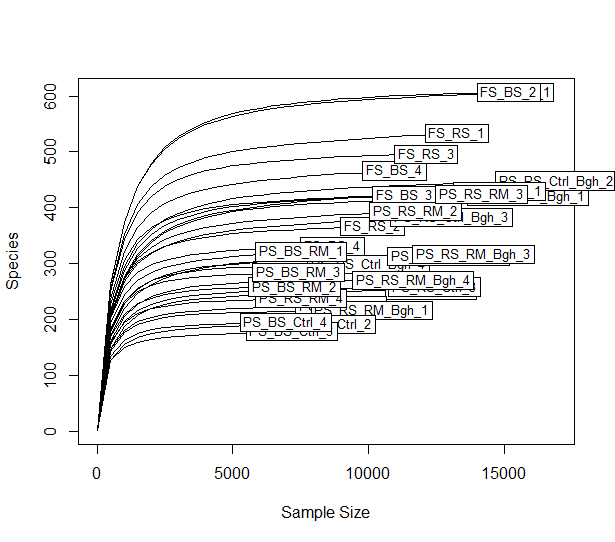


## Supplementary Tables

Supplementary Table 1. Pielou’s evenness, Species richness and Shannon diversity index of barley rhizosphere and bulk soil samples, either with control (Ctrl) or rhizosphere microbiome transplant (RMT) treatment and unchallenged (-*Bgh*) or *Bgh* challenged (+*Bgh*). Different letters indicate significant differences (Tukey’s test *p* ≤ 0.05).

|  | Rhizosphere | | | | | Bulk soil | |
| --- | --- | --- | --- | --- | --- | --- | --- |
|  | Ctrl | | | RMT | | Ctrl | RMT |
|  | -*Bgh* | +*Bgh* | | -*Bgh* | +*Bgh* |  | |
| **Pielou's evenness** | 0.86±0.02b | | 0.87±0.01ab | 0.89±0.01a | 0.90±0.01a | 0.85±0.01b | 0.91±0.01a |
| **Species richness** | 240.45±16.14b | | 273.54±43.61ab | 359.27±82.92ab | 373.95±57.11a | 187.23±8.48b | 286.83±30.88a |
| **Shannon index** | 4.69±0.10c | | 4.88±0.18bc | 5.23±0.24ab | 5.30±0.11aa | 4.44±0.07b | 5.15±0.12a |

Supplementary Table 2. Taxonomic affiliation of *Micrococcaceae* ASVs that were not classified on genus level by 16S rRNA gene amplicon sequencing.

| **ASV ID** | **Taxonomic affiliation according to NCBI (sequence identity)** | **Number of total reads** |
| --- | --- | --- |
| Family_Micrococcaceae_1 | *Pseudarthrobacter phenanthrenivorans* (100%) or *Arthrobacter humicola* (100%) | 5024 |
| Family_Micrococcaceae_2 | *Pseudarthrobacter scleromae* (100%) or *Arthrobacter globiformis* (100%) | 2344 |
| Family_Micrococcaceae_3 | *Arthrobacter bambusae* (100%) | 367 |
| Family_Micrococcaceae_4 | *Arthrobacter gyeryongensis* (100%) or *Arthrobacter methylotrophus* (100%) | 249 |
| Family_Micrococcaceae_5 | *Pseudarthrobacter phenanthrenivorans* (99.75%) or *Arthrobacter humicola* (99.75%) | 49 |
| Family_Micrococcaceae_6 | *Pseudarthrobacter phenanthrenivorans* (99.75%) or *Arthrobacter humicola* (99.75%) | 25 |
| Family_Micrococcaceae_7 | *Pseudarthrobacter phenanthrenivorans* (99.29%) | 4 |
| Family_Micrococcaceae_8 | *Arthrobacter psychrochitiniphilus* (89.78%) | 2 |

Supplementary Table 3. Relative abundance of 20 taxa differing between the rhizosphere microbiome transplant (RMT) and the control (Ctrl) in either the unchallenged or *Bgh*-challenged samples or both. Mean ±SD. Bold numbers represent the significant differences determined by edgeR.

| **Taxon** | **Ctrl** | **Ctrl +*Bgh*** | **RMT** | **RMT +*Bgh*** |
| --- | --- | --- | --- | --- |
| *Pedobacter* | 10.58 ±2.22 | **13.92 ±1.72** | 9.10 ±1.02 | **8.01 ±0.50** |
| *Rhizobium* | **8.71 ±0.75** | 9.00 ±1.79 | **4.22 ±0.72** | 6.16 ±2.91 |
| *Pseudomonas* | **8.76 ±5.49** | 5.25 ±1.29 | **3.79 ±0.53** | 4.38 ±0.65 |
| *F Burkholderiaceae* | **8.66 ±1.01** | **5.19 ±1.15** | **1.54 ±0.70** | **2.07 ±0.44** |
| *Exiguobacterium* | 4.81 ±4.59 | **5.71 ±1.73** | 4.32 ±3.86 | **1.49 ±0.45** |
| *Flavobacterium* | 3.78 ±0.34 | **2.17 ±0.58** | 4.71 ±0.78 | **4.54 ±0.68** |
| *Bacillus* | **5.82 ±2.13** | 4.20 ±0.90 | **2.32 ±0.93** | 2.85 ±0.38 |
| *Paenibacillus* | **5.93 ±1.19** | **4.80 ±1.31** | **2.10 ±0.43** | **1.82 ±0.52** |
| *Devosia* | 4.11 ±2.10 | **4.48 ±0.49** | 2.93 ±0.42 | **2.40 ±0.37** |
| *Dyadobacter* | **5.00 ±1.31** | **4.01 ±1.10** | **2.03 ±0.52** | **2.41 ±0.46** |
| *F Micrococcaceae* | **0.09 ±0.16** | **0.08 ±0.15** | **4.80 ±0.57** | **4.50 ±1.29** |
| *Fluviicola* | **0.47 ±0.27** | 1.87 ±1.09 | **2.89 ±0.45** | 2.37 ±0.50 |
| *F Rhizobiaceae* | **0.64 ±0.55** | **0.59 ±0.20** | **2.64 ±0.35** | **2.44 ±0.29** |
| *Brevundimonas* | **1.71 ±0.82** | 2.20 ±0.57 | **0.74 ±0.19** | 1.17 ±0.15 |
| *O Saccharimonadales* | 0.06 ±0.11 | **0.08 ±0.09** | 1.90 ±0.40 | **3.09 ±2.49** |
| *Azospirillum* | **3.66 ±3.16** | **1.84 ±0.06** | **0.31 ±0.11** | **0.16 ±0.05** |
| *F Moraxellaceae* | 0.67 ±0.19 | **0.61 ±0.24** | 0.59 ±0.38 | **1.95 ±1.83** |
| *F Caulobacteraceae* | **0.01 ±0.01** | **0.04 ±0.07** | **1.26 ±0.35** | **1.63 ±0.07** |
| *F Cytophagaceae* | **0.00 ±0.00** | **0.20 ±0.09** | 0.20 ±0.09 | 0.02 ±0.03 |
| *Emticicia* | **0.00 ±0.00** | **0.08 ±0.02** | 0.10 ±0.03 | 0.09 ±0.07 |

Supplementary Table 4. Taxonomic affiliation and *in vitro* functional characteristics of bacterial isolates obtained from the rhizosphere microbiome transplant.

| Isolate | Taxonomic affiliation according to NCBI database(% identity) | Protease | β-1,3-Glucanase | Cellulase | Chitinase | ACC deaminase | Siderophore | Indole-3-acetic acid | Short-chain AHL | Long-chain AHL | Phosphate solubilization Solubilation |
| --- | --- | --- | --- | --- | --- | --- | --- | --- | --- | --- | --- |
| 1 | *Microbacterium* sp. (99.48%) | - | - | - | - | + | - | + | - | - | - |
| 2 | *Brevibacterium* sp. (98.54%) | + | - | - | - | - | - | - | - | - | - |
| 3 | *Pantoea* sp. (98.28%) | - | - | - | - | - | + | + | + | - | + |
| 4 | *Arthrobacter* sp. (99.88%) | + | + | - | - | + | (+) | - | - | - | - |
| 5 | *Mucilaginibacter* sp.(98.45%) | - | - | + | - | - | - | + | - | - | - |
| 6 | *Flavobacterium* sp. (97.82%) | + | (+) | + | - | - | + | - | - | - | - |
| 7 | *Pseudarthrobacter* sp. (99.88%) | + | - | + | - | + | - | + | - | - | - |
| 8 | *Microbacterium* sp. (99.47%) | - | - | - | + | - | - | + | - | - | - |
| 9 | *Bacillus* sp. (99.06%) | + | - | - | - | - | - | - | - | - | - |
| 10 | *Curtobacterium* sp. (99.41%) | + | + | - | - | - | + | + | - | - | - |
